# Wnt16 signaling promotes osteoblast differentiation of periosteal derived cells in vitro and in vivo

**DOI:** 10.7717/peerj.10374

**Published:** 2020-11-24

**Authors:** Ying Jin, Xiaoyan Sun, Fang Pei, Zhihe Zhao, Jeremy Mao

**Affiliations:** 1 Department of Orthodontics, State Key Laboratory of Oral Diseases, West China Hospital of Stomatology, West China School of Stomatology, Sichuan University, Chengdu, China; 2 Stomatological Center, the First Affiliated Hospital of Zhengzhou University, Zhengzhou, China; 3 Columbia University, Center for Craniofacial Regeneration, New York, NY, United States of America

**Keywords:** Periosteum derived cell, Osteogenesis, β-TCP, β-catenin

## Abstract

**Background:**

Periosteum plays critical roles in de novo bone formation and fracture repair. Wnt16 has been regarded as a key regulator in periosteum bone formation. However, the role of Wnt16 in periosteum derived cells (PDCs) osteogenic differentiation remains unclear. The study goal is to uncover whether and how Wnt16 acts on the osteogenesis of PDCs.

**Methods:**

We detected the variation of Wnt16 mRNA expression in PDCs, which were isolated from mouse femur and identified by flow cytometry, cultured in osteogenic medium for 14 days, then knocked down and over-expressed Wnt16 in PDCs to analysis its effects in osteogenesis. Further, we seeded PDCs (Wnt16 over-expressed/vector) in *β*-tricalcium phosphate cubes, and transplanted this complex into a critical size calvarial defect. Lastly, we used immunofluorescence, Topflash and NFAT luciferase reporter assay to study the possible downstream signaling pathway of Wnt16.

**Results:**

Wnt16 mRNA expression showed an increasing trend in PDCs under osteogenic induction for 14 days. Wnt16 shRNA reduced mRNA expression of Runx2, collage type I (Col-1) and osteocalcin (OCN) after 7 days of osteogenic induction, as well as alizarin red staining intensity after 21days. Wnt16 also increased the mRNA expression of Runx2 and OCN and the protein production of Runx2 and Col-1 after 2 days of osteogenic stimulation. In the orthotopic transplantation assay, more bone volume, trabecula number and less trabecula space were found in Wnt16 over-expressed group. Besides, in the newly formed tissue Brdu positive area was smaller and Col-1 was larger in Wnt16 over-expressed group compared to the control group. Finally, Wnt16 upregulated CTNNB1/*β*-catenin expression and its nuclear translocation in PDCs, also increased Topflash reporter luciferase activity. By contrast, Wnt16 failed to increase NFAT reporter luciferase activity.

**Conclusion:**

Together, Wnt16 plays a positive role in regulating PDCs osteogenesis, and Wnt16 may have a potential use in improving bone regeneration.

## Introduction

Periosteum, a bilayer fibrous soft tissue, is fundamental to both intramembranous bone formation and endochondral ossification during skeletal development and natural bone fracture healing (Colnot, Zhang & Tate, 2012; Debnath et al., 2018). Outer layer of periosteum comprises of fibroblasts and extracellular matrix, while inner layer acts as the reservoir of progenitor cells and osteoblasts (Colnot, Zhang & Tate, 2012). Recent decades, periosteum as a central mediator of bone healing has become an optimal cell source for tissue engineering. Periosteum derived cells (PDCs), isolated from periosteum with an elevator and digested with enzyme, have been proved to possess highly proliferation ability and mesenchymal multipotency(osteogenic, chondrogenic, adipogenic and myogenic) (Roberts et al., 2015). PDCs contain plenty of stem cells that continuously give rise to osteoblasts, even in elderly patients (Evans et al., 2013; Ferretti et al., 2015; Ferretti & Mattioli-Belmonte, 2014; Uematsu et al., 2013). Those findings generate immense enthusiasm in its utilization in bone tissue regeneration. But its osteogenic and bone regenerative abilities are not well studied.

Wnt16 has been regarded as a key regulator in bone metabolism. Osteoblast-specific overexpression of Wnt16 increases both cortical and trabecular bone mass and structure in mice (Alam et al., 2016). In addition, Wnt16 conditionally ablated mice showed reduced cortical bone thickness, and it was attributed to increased bone resorption and reduced periosteal bone formation (Ohlsson et al., 2018). Another study revealed that the periosteal bone formation rate and mineral apposition rate were reduced in Wnt16 knockout vs wild-type mice (Wergedal et al., 2015). Thus, Wnt16 may be a key player in femoral periosteum bone formation. However, the exact role of Wnt16 in PDCs osteogenic differentiation and its utilization in bone regenerative applications remain unclear.

Together, our goal was to discover the function of Wnt16 in PDCs, as well as the capability of Wnt16 overexpressed-PDCs in bone regeneration in a critical size bone defect model.

## Materials & Methods

### Isolation and culture of PDCs

This study was approved by the Research Ethics Committee of West China Hosipital of Stomatology Sichuan University, PR China. (No. WCHSIRB-D2018-093). The CD-1 mice were purchased from Animal experiment center of Sichuan University. Animals acclimated for at least a week from the day of arrival to day of surgical procedure. Animals were euthanized via CO2 inhalation followed by cervical dislocation. Adherent cells were isolated from femoral periosteum of postnatal 4 (P4) weeks CD-1 mice. PDCs were prepared by three sequential enzymatic digestions of femur. Femur were digested with 3 mg/mL of collagenase type I and 4 mg/mL of dispase (Invitrogen, Carlsbad, CA, USA), for 75 min at 37 °C, followed by passage through a 70-µm strainer (BD, Franklin Lakes, NJ, USA). The first digestion (15 min) was discarded as to prevent interference with the connective tissue. Cells from digestions II to III (30min each) were plated in T-75 flasks. PDCs were isolated and cultured in low-glucose Dulbecco’s modified Eagle’s medium (DMEM, Gibco, USA) containing 20% fetal bovine serum (FBS; Gibco BRL). Passage 3 were used for all in vitro and in vivo experiments in consideration of a balance to obtain sufficient cells for study but also without extended culture that is associated with the loss of cellular phenotypes.

### Flow cytometry

Flow cytometric analysis was used to evaluate the expression of CD105, stem cell antigen-1 (Sca-1), CD90 and CD45. Passage 3 PDCs were incubated individually and simultaneously with monoclonal mouse anti-mouse fluorochrome-conjugated antibodies for 30 min at room temperature. The antibodies were APC-CD105-647 (Cat. No. 120413, Rat anti-Mouse, Biolegend), PE-Cy7-Sca-1 (Cat. No. 108113, Rat anti-Mouse, Biolegend), FITC-CD90.2 (Cat. No. 140303, Rat anti-Mouse, Biolegend) and PE-CD45 (Cat. No. 400607, Rat anti-Mouse, Biolgegend). After washing with 3% PBSF (3%FBS in phosphate buffer saline) twice, the samples were evaluated with 4-color Beckman Coulter FC 500.

### Real-time quantitative PCR

Total mRNA of PDCs was extracted using TRIzol (Invitrogen). cDNA synthesis was done by reverse transcription of total RNA with TaqMan reverse transcription reagents (Applied Biosystems, CA, USA). Target gene expression was quantified using TaqMan Gene specific primers and normalized to glyceraldehyde 3-phosphate dehydrogenase (GAPDH) by using ViiA 7 Real-Time PCR System (ThermoFisher, USA). The following primer/probe sets were used: mouse Wnt16, Mm00437347_m1; mouse Runx2, Mm00501584_m1; mouse osteocalcin/BGLAP (Ocn), Mm03413826_mH; Col-1/Co1a1, Mm00801666_g1 (Applied Biosystems, CA, USA).

### RNA interference and overexpression

We used shRNA to knock down the expression of Wnt16 in PDCs. Liposome encapsulated PLKO.1 RiWnt16/PLKO.1 Scramble, PVSVG, Pdeta8.9 was transfected into 80% confluent 239T cells, Virus supernatant was collected 2 days after transfection. PDCs were infected with the virus supernatant, and the stable positive cell lines were filtrated by using puromycin (1µl, Gibco, USA). Control pHBLV-CMV-EF1-RFP or Wnt16 overexpressed (OE) pHBLV-CMV-EF1-RFP-Wnt16 (6.25 mg), pMD2.G (Plasmid #12259, Addgene) (0.625 mg) and psPAX2 (Plasmid #12260, Addgene) (3.125 mg) vectors were co-transfected into 293T cells using Calcium Phosphate Transfection Kit per manufacturer’s protocol (Invitrogen). Virus supernatant was filtered with 0.45 mm membrane and purified with ViraBind™ Lentivirus Purification Kit (Cell Biolabs). PDCs were infected with lentivirus in 8 mg/mL polybrene (Santa Cruz Biotechnology). Infected cells and non-infected cells (blank group) were sorted by FACS based on RFP expression (Figs. S1A–S1E) and were further passaged 3–5 times.

### Cell proliferation and differentiation

Control and Wnt16 knocking down PDCs were seeded in 96-well plates at a density of 3 × 103 cells per well. Cell numbers were analyzed on day 1, 2 and 3 by Cell Counting Kit-8 (CCK-8) (HY-K0301, MCE, USA) at 450-nm absorbance. For osteogenic differentiation, control and Wnt16 knocking down or overexpressed PDCs were seeded at 70% confluence and cultured with DMEM Medium for 24 h before switched the osteogenic differentiation medium consisting of 100 mM ascorbic acid, 2 mM b-glycerophosphate, 10 nM dexamethasone, with medium change every 2 days. RNA samples were collected at day 0, 2, 3, 7 and 14 for gene expression analysis and protein samples were collected at day 4 for western blotting. Alkaline phosphatase (ALP) staining (Stemgent) was performed at day 7, and Alizarin red staining at day 21.

### In vivo cells transplantation

We used porous *β*-tricalcium phosphate (*β*-TCP) scaffold (5 × 2 mm^3^; pore size: 200–500 µm; porosity: 50–70%), which was produced by the National Engineering Research Center for Biomaterials, Sichuan University. As we reported previously, 1 × 10^6^ control or Wnt16 over-expressed PDCs were planted on surfaces of each scaffold and were primed for osteogenic differentiation at 37 °C and 5% CO2 for 2 days (Zhou et al., 2015). 27 CD-1 mice (male, 4–5 wks, 6 mice per group) were used in the in-vivo experiments. These mice were randomly distributed into three groups: blank, control and Wnt16 OE. The mice were first anesthetized with isoflurane. Calvarial defect (diameter: 5 mm) were created on the calvarium of mouse by using a circular hollow bur, and the disconnected cranium was carefully lifted without destroying the underneath blood vessels. Both control and Wnt16 over-expressing PDCs were seeded on *β*-TCP scaffolds. Scaffolds without cells (blank group) and with control or Wnt16 OE cells were implanted into a calvarial defect. Upon recovery, animals were closely monitored every 2–3 mins until they maintained a sternal position. Animals were feed on soft food for next one week post-operatively. Animals continued to be observed twice daily for next 3 days post-operatively and every other day thereafter. If signs such as hunched posture, lethargy, lack of food intake, skin irritation or infection of the surgery site, dehiscence of the incision site are observed, a veterinarian would be contacted or the mouse would be euthanized by CO2 followed by cervical dislocation.

### Micro-CT imaging analyses

Un-decalcified calvaria samples were harvested and fixed with 4% paraformaldehyde, then scanned using a micro-computed tomography (micro-CT) system (Scanco Medical, Bassersdorf, Germany) at a voxel size of 10.5 µm3 to image bone. The cranium image was reconstructed via MicroView ABA 2.2 software. The regions of interest (ROIs) were bone like structure in the defect. New bone formation rate was identified as the trabecular bone volume (BV)/scaffold tissue volume (TV). The quantitative structural parameters were trabecular number (TbN, 1/mm) and trabecular space (TbSp, µm).

### Histology

Eight weeks later, animals were euthanatized by CO2 followed by cervical dislocation, the transplants were collected and fixed with 4% paraformaldehyde. After fixation, samples were demineralized in 0.5 M EDTA (pH 7.4) for 1 week and then dehydrated. Paraffin sections (5 mm) were used for hematoxylin-eosin (HE), Masson’s trichrome and immunohistochemical staining. We used Anti-Mouse HRP-DAB Cell & Tissue Staining Kit (R & D Systems) in the immunohistochemical staining. After deparaffinizing, heat-retrieving and blocking, sections were incubated with the primary antibodies (Brdu, ab270260, Abcam; Col11A1, ab64883, Abcam) overnight at 4 °C, then incubated with HRP conjugated secondary antibodies. Finally, images were developed with DAB. We stained three tissue sections per sample. The ROIs were calcified region, and we randomly selected three fields (400X). The BrdU and Col1 were analyzed by their integrated optical density (IOD) value via image pro plus 6.0 software. When we calculated the IOD, we eliminated the color “blue” interference which stands for the nucleus.

### Western blot

After washing with ice-cold PBS, protein was extracted by using RIPA Lysis Buffer (Thermo Scientific) with Protease/Phosphatase Inhibitor Cocktail (ab201119, Abcam USA). Samples were added on a NuPAGE® Novex® 4e12% BiseTris Protein Gel (1.0 mm), transferred to nitrocellulose membrane, and incubated with anti-Pro-Col-1 (sc-8782, 1:200, Santa Cruz Biotechnology), and anti-GAPDH (sc-25778, 1:200, Santa Cruz Biotechnology) antibodies. Following using corresponding secondary antibodies, image was developed with IR fluorescence & Odyssey.

### Immunohistochemistry

For immunocytochemical staining, control and Wnt16 overexpressed PDCs were cultured in the 6 well cell culture cluster until getting 70–80% confluence. After blocking, cells were incubated with anti-CTNNB1 antibody (ab32572, 1:200, Abcam) overnight at 4 °C.

### Transfections and luciferase reporter activity

We seeded PDCs into 24-well plates and transiently transfected them with Topflash (Plasmid,12457, Addgene) + Wnt16 (Plasmid, 42291, Addgene)/NFAT (Plasmid 11792, Addgene) + PGL4 [hRluc/sv40] (Plasmid, 64034, Addgene) and Topflash+ EGFP (Plasmid, 54762, Addgene) + PGL4 as control using Neon® Transfection System for Electroporation (Thermo Fisher Scientific) according to the manufacturer’s protocol. Post-transfection, cells were starved for 8 h, and then 50 ng/ml rhDKK1 (R&D Systems) or 50 ng/ml rhWnt3a (Abcam) or 50 ng/ml rhWnt5a (Abcam) or 5,000 ng/ml INCA-6 (R&D Systems) was used to assay canonical Wnt and non-canonical Wnt pathways. After 16 hours‘ incubation, Cells were lysed and luciferase activities were measured according to the manufacturer’s instructions (Luciferase Reporter Assay System; Promega, CA, USA).

### Statistical analysis

Experiments were performed in triplicate. We used SPSS software for our analyses. Quantitative data were analyzed for statistical difference using Student’s *t*-test or one-way ANOVA, with significance of *p* < 0.05. The values are expressed as mean ± SD.

## Results

### PDCs isolation and morphological characterization

**Figure 1:**
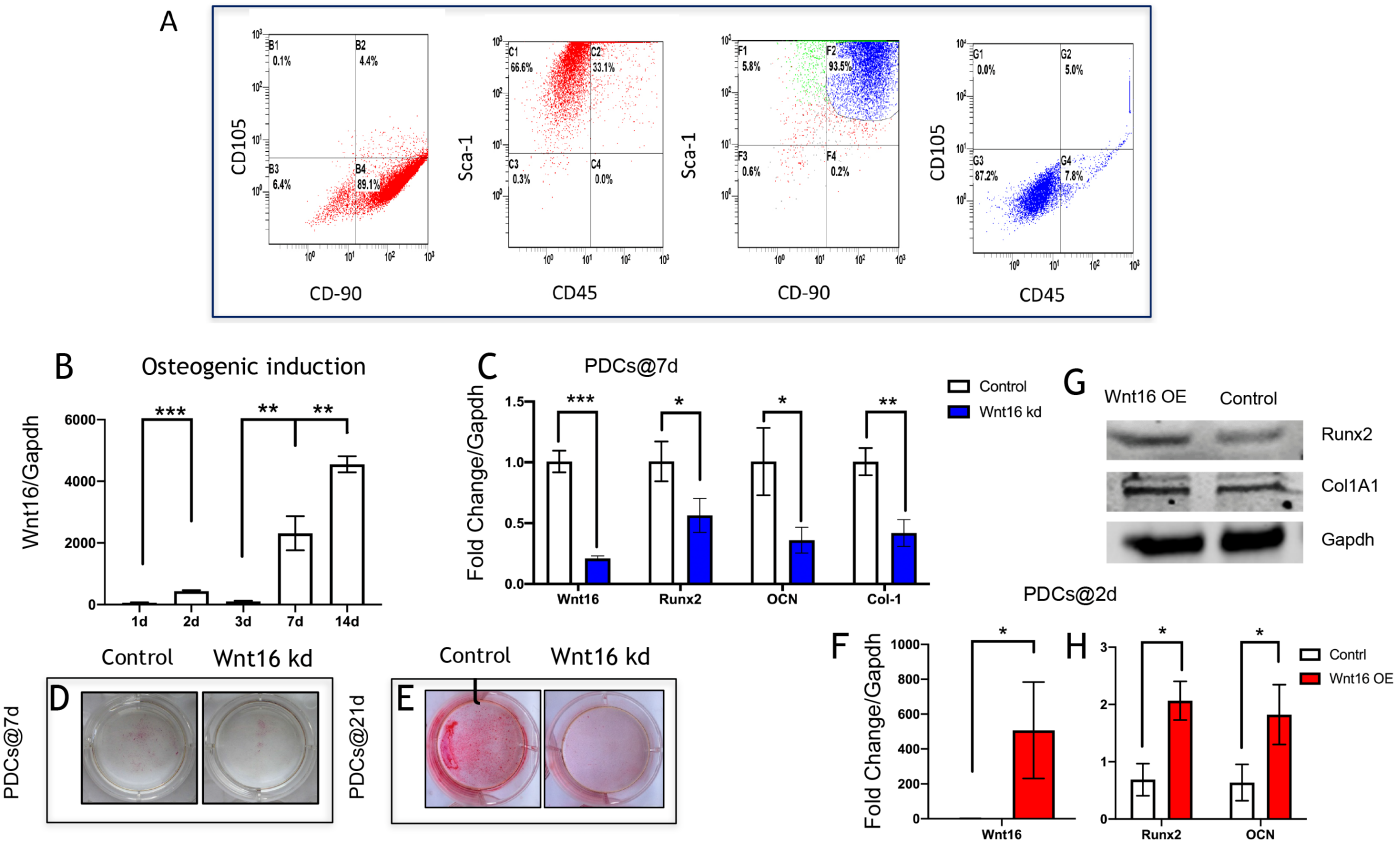
Wnt16 expression and function in osteogneisis of periosteum derived cells (PDCs). Wnt16 expression and function in osteogneisis of periosteum derived cells (PDCs). (A) Flow cytometry showed that approximately 89.1% in the third passage of the isolated PDCs were CD90^+^/CD105^-^, and approximately 66.6% of them were Sca-1^+^/CD45^-^. Within CD90^+^/Sca-1^+^, 87.2% cells wereCD90^-^/CD45^-^. (B) Change of Wnt16 mRNA expression in PDCs under osteogenesis induction medium for 14 days (*n* = 3). (C) mRNA of Wnt16, Runx2, OCN and collagen types I (Col-1) after Wnt16 knocking down (kd) by using wnt16 shRNA in PDCs after 7 days of osteogenic indction (*n* = 3). (D) Whole plate mount of alkaline phosphataseactivity in PDCs with or without Wnt16 knocking down after 7 days of osteogenic indction. (E) Whole plate mount of Alizarin Red staining image in PDCs after 21 days of induction. mRNA of Wnt16 (F), Runx2 and OCN (H) upon Wnt16 overexpression (OE) by using lentivirus vector after 2 days of induction. (*n* = 3) (G) Runx2 and Col-1 protein upon Wnt16 overexpression. * *p* < 0.05, ** *p* < 0.001, *** *p* < 0.0001.

PDCs are typically identified as mononucleated adherent cells isolated from femoral periosteum, which are thought to contain plenty of MSCs (Debnath et al., 2018; Ferretti & Mattioli-Belmonte, 2014). The cell surface antigens were characterized by flow cytometry analyses. Flow cytometry revealed that 89.1% cells were CD90+CD105-, and 66.6% cells were Sca-1+CD45-. In addition, within Sca-1+CD90+, there were 87.2% cells were CD105-CD45-(Ferroni et al., 2019; Ng et al., 2020) (Fig. 1A). These results revealed that these isolated cells from femoral periosteum contain a proportion of stem cells.

### Wnt16 involves in PDCs osteogenesis

We analyzed the mRNA of Wnt16 at day 0, 2, 3, 7 and 14 days to uncover the expression pattern of Wnt16 during the osteogenesis of PDCs. We cultured the PDCs in osteogenic medium for 14 days, and detected Wnt16 mRNA expression via qPCR. Wnt16 mRNA expression up-regulated significantly in PDCs at day 7 and 14 (*p* < 0.0001) (Fig. 1B). In order to analyze the role of Wnt16 in the ostegenesis of PDCs, Wnt16 shRNA or Vector was transfected into PDCs. Endogenous Wnt16 was reduced to ∼1/8 of the original level in PDCs (*p* < 0.0001) (Fig. 1C). Our CCK-8 analysis resulted Wnt16 shRNA transfection did not affect the proliferation rate of those cells (Fig. S1), suggesting perhaps its restricted roles in regulating mesenchyme differentiation and maturation without necessarily affecting cell growth. Because the expression of Wnt16 increased dramatically from day 7, so we checked the change of the mRNA expression of osteogenic transcriptional factors/genes, such as Runx2, collage type I (Col-1), and osteocalcin (Ocn) after osteogenic induction for 7 days. Interestingly, Wnt16 shRNA reduced significantly the mRNA expression of Runx2, Col-1 and Ocn (*p* < 0.05). (Fig. 1C) In addition, ALP staining intensity was also diminished a little in Wnt16 knocked down PDCs. (Fig. 1D) Surprisingly, after 21 days‘ostegenic induction Wnt16 shRNA dramatically weakened the intensity of alizarin red staining, stands for the mineralization of the extracellular matrix (Aguila & Rowe, 2005). (Fig. 1E) Besides using shRNA to knock down Wnt16 expression, we used lentivirus infection to overexpress Wnt16 in PDCs to uncover whether up-regulated Wnt16 would promote the early process of osteogenesis. Wnt16 was enhanced to 8 times of the original level (*p* < 0.0001) (Fig. 1F), which significantly promoted not only mRNA expression (*p* < 0.05) (Fig. 1H) but also protein production (Fig. 1G) of Runx2 after two days of osteogenic stimulation. Meanwhile, OCN mRNA expression (*p* < 0.05) and Col-1 protein production were also enhanced by Wnt16 overexpression. Taken together, those data indicate that Wnt16 may up-regulate osteoblast differentiation and mineralization during PDCs osteogenesis.

### Wnt16 enhances collagenous fibers formation in critical size calvarial bone defect

**Figure 2:**
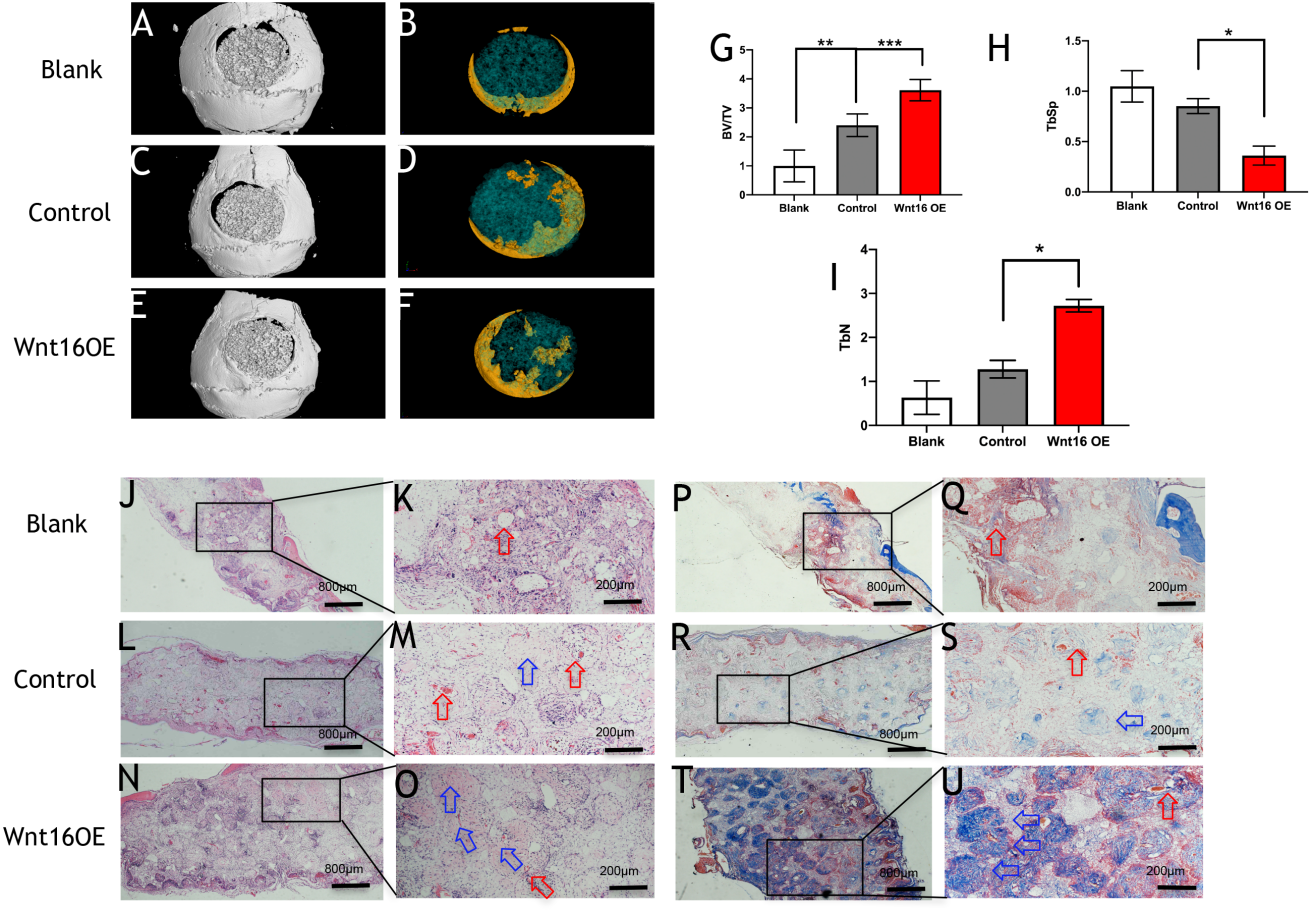
Wnt16 enhanced mineralized tissue formation in vivo by PDCs/ *β*-TCP implantation. (A–F) Representative micro-CT images of bone formation. Yellow stands for newly formed bone like tissue. (G–I) Quantitative analysis of trabecular bone volume (BV)/scaffold tissue volume (TV) ratio, trabecular space (Tb.Sp), trabecular number (Tb.N). (J–Q, P–U) Histological analysis of bone formation at 8 weeks after implantation: H & E staining (J–Q); Masson’s trichrome stainings (P–U). (J, L, N, P, R, T) Magnification × 100; (K, M, O, Q, S, U) Magnification × 400; Red arrow: blood vessel; Blue arrow: mineralized matrix. Scale bar: 200 µm. * *p* < 0.05, ** *p* < 0.001, *** *p* < 0.0001.

**Figure 3:**
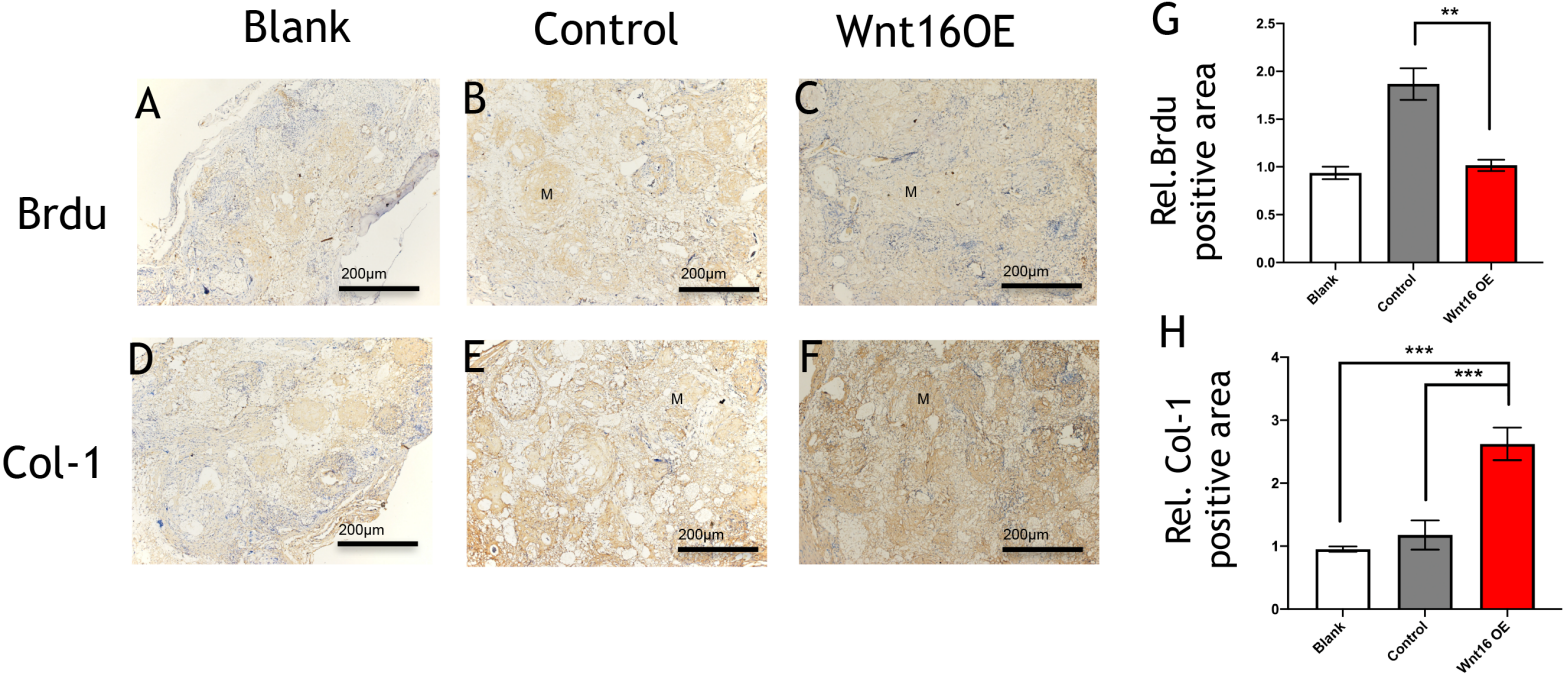
Immunohistological staining of MSCs and osteogenesis marker proteins in calvarial defects at 8 weeks after surgery. (A–C) Brdu production in mouse calvarial defects. (G) Brdu expression was significantly lower than the control group (*n* = 3). (D–F) Col-1production in mouse calvarial defects. (H) Col-1 expression was significantly higher than the control group (*n* = 3). OE, over-expressed; m, matrix, Scale bar: 200 µm. * *p* < 0.05.

To further investigate the possibility of using Wnt16 altered-PDCs in bone regeneration, we established a mouse critical size calvarial defect. The genetically engineered PDCs was embedded in *β*-TCP scaffold. Then, we transplanted those graft into a calvarial defect (diameter: 5 mm). After 8 weeks of healing, we studied the capacity of calvarial defect restored and the quantity of newly formed bone in the defect sites by micro-CT. New bone formation was observed not only in the marginal but also in central regions of the defects in Wnt16 overexpression (OE) groups. (Figs. 2A–2F) Morphometric analysis of the quantity of newly formed bone indicated that more BV/TV, TbN and less TbSp were found in Wnt16 OE group (*p* < 0.05). (Figs. 2G–2I) In addition, the histological characteristics were observed in all groups. Blood vessel was observed in all groups, without any significant difference among groups. In HE and Masson staining images, abundant extracellular matrix and collagen was observed in Wnt16 OE groups. (Figs. 2J–2O & 2P–2U). In addition, Brdu positive area was attenuated by Wnt16 OE (Figs. 3A–3C, 3G), but Col-1 was more intensive in Wnt16 OE group than in control and blank groups. (Figs. 3D–3F, 3H). Taken together, our data suggest that Wnt16 promoted the transplanted cells undergoing osteogenic differentiation, leading to more collagenous fibers formation in the calvarial bone defect.

### Wnt16 activating canonical *β*-catenin pathway

**Figure 4:**
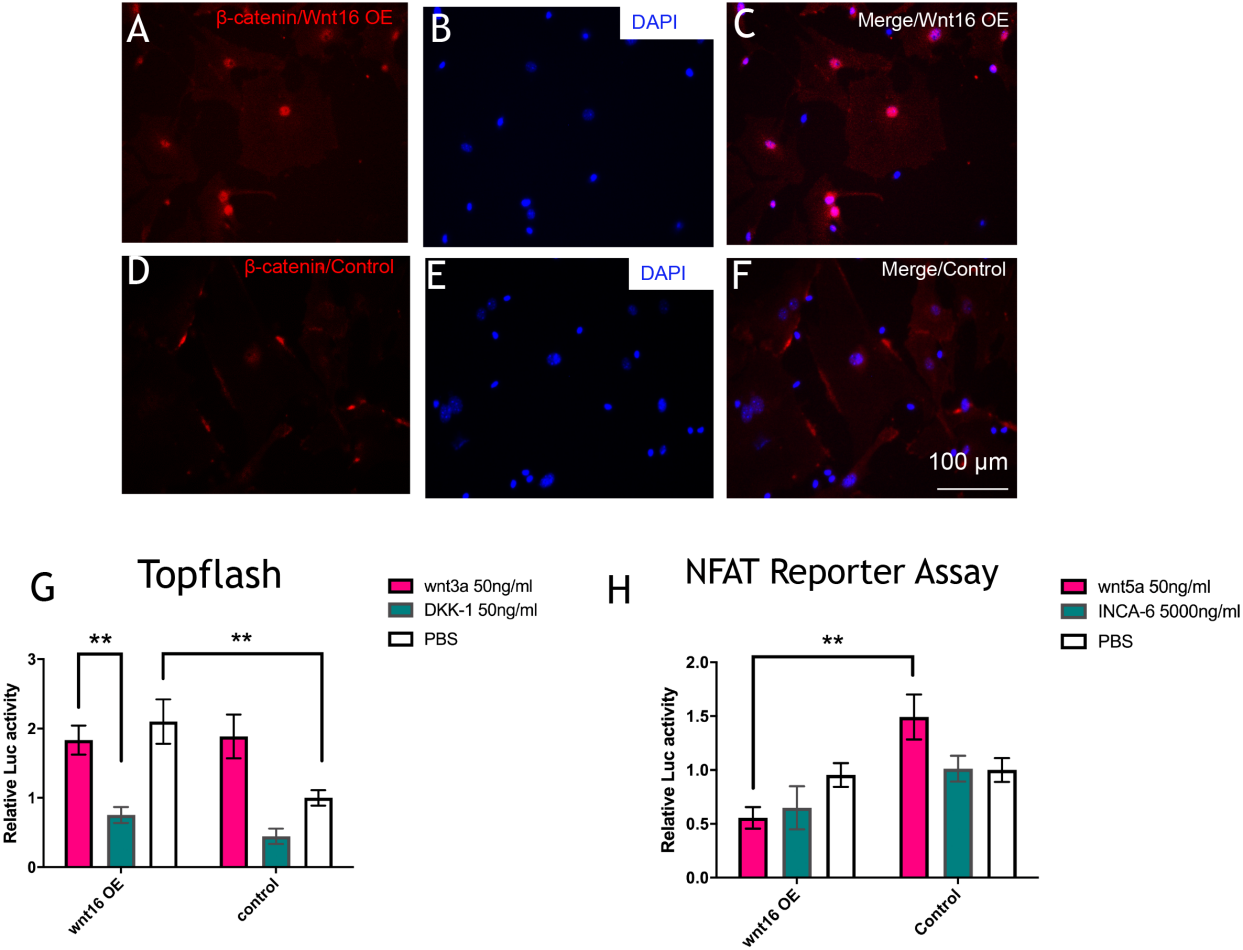
Wnt16 activate canonical wnt signaling pathway. (A–F) immunofluorescence staining of *β*-catenin in Wnt16 oeverxpressed (OE) PDCs or in control groups. Overexpression of Wnt16 in PDCs promote *β*-catenin translocating into nucles. (G) TCF/LEF promoter relative activity was quantified by transfecting the TCF/LEF reporter and using a luciferase assay in Wnt16 OE cells added with PBS, Wnt3a or DKK-1 (*n* = 3). (H) NFAT-dependent transcriptional activity was quantified by transfecting the NFAT reporter and using a luciferase assay in Wnt16 OE cells added with PBS, Wnt5a or INCA-6 (*n* = 3). Scale bar: 100 µm, * *p* < 0.05.

To further understand the molecular mechanisms of Wnt16 regulation of osteogenic differentiation, we used Wnt16 OE PDCs to test the potential downstream signaling factors, which are *β* -catenin and WNT/Ca2+. Strikingly, immunofluorescence staining revealed that Wnt16 promoted CTNNB1 expression and nuclear translocation (Figs. 4A–4F). Consistent with the increased nuclear translocation of CTNNB1, Wnt16 OE further increased the TCF/LEF reporter activity in a Topflash assay (*p* < 0.05), which indicates Wnt/*β*-catenin signaling pathway activation (Ye et al., 2019). But Wnt16 OE along with Wnt3a, a canonical Wnt that is thought to activate Wnt/*β*-catenin signaling pathway, showed almost similar effect when Wnt16 OE or Wnt3a existed alone; meanwhile DKK-1, a specific inhibitor of the target of the Wnt/*β*-catenin pathway, restrained Wnt16‘s effect (Fig. 4G) (Baron & Kneissel, 2013). On the other hand, Wnt16 OE failed to increase NFAT reporter activity, which indicates the activation of calcineurin/Ca2+ pathway (Li et al., 2016). However, when pretreatment with Wnt5a, a noncanonical wnt that has been proved to activate NFAT transcription factors, Wnt16 reduced the relative luciferase activity compared to the control group (*p* < 0.05) (Fig. 4H). In addition, no significantly effect was found when adding a calcineurin-NFAT interaction inhibitor INCA-6. Taken together, our data suggests that Wnt16 activates *β*-catenin signaling, but does not trigger off Wnt/Ca2+ pathway, and even may be a competitive inhibitor to Wnt5a regarding to the activation of Wnt/Ca2+ pathway.

## Discussion

Wnt16 has been recognized as a positive regulator of cortical bone thickness and periosteal bone formation (Alam et al., 2016; Ohlsson et al., 2018; Wergedal et al., 2015). Our findings elaborate that Wnt16 appears to effect the whole stages of osteogenesis in periosteal derived cells (PDCs). Firstly, we identified the existence of stem cells in the PDCs. Flow cytometry showed 50.6% cells in the PDCs were Sca-1+/CD45-, and 51.9% cells were CD105-/34-. Secondly, under the induction of osteogenic medium, Wnt16 increased dramatically in PDCs from day 7 to day 14. Knocking down of Wnt16 in PDCs significantly reduced Runx2, Ocn and Col-1 expressions, while Wnt16 overexpression promoted both Runx2 mRNA expression and protein production. In addition, knocking down of Wnt16 has a tendency to reduce ALP activity, an earlier osteoblast differentiation marker, and dramatically reduced the alizarin red staining intensity, which refers to the later mineralization stage of osteogenesis (Mortada & Mortada, 2018). Thirdly, using a model of bone injury, PDCs genetically engineered to express Wnt16 significantly regenerated more bone-like tissues in the critical size bone defects in vivo. Finally, we confirm that Wnt16 activates canonical Wnt *β*-catenin signaling, which is consistent with the previous studies (Hendrickx et al., 2020; Wergedal et al., 2015). However, Wnt16 did not trigger off Wnt/Ca2+ pathway, but may indirectly inhibit Wnt/Ca2+ pathway by antagonizing Wnt5a. The present findings suggest that Wnt16 act positively in PDCs osteogenesis process, and Wnt16 modified PDCs may be promising for bone regeneration strategies.

Recently, periosteal cells have generated immense enthusiasm regarding its utilization in tissue engineering and regeneration due to its essential roles in periosteum mediated de novo bone formation and fracture repair (Bragdon & Bahney, 2018; Debnath et al., 2018; Yang et al., 2020). However, periosteal cell cultures have indicated the heterogeneous nature of PDCs with MSCs, fibroblasts and osteogenic cells (Gao et al., 2019). Previous study proved that bone-derived Sca-1+/CD45-/CD31- cells exhibiting trilineage osteoblastic, adipocytic, and chondrocytic differentiation ability and higher clonogenic efficiencies (Holmes & Stanford, 2007). Interestingly, in our isolated PDCs, 66.6% cells were Sca-1+/CD45- which indicates that half of the PDCs probably possess pluripotency capability. Furthermore, Debnath et al. identified three populations among periosteum derived cells, which were CD49^flow^/CD51^low^: CD200+/CD105- periosteal mesenchymal stem cells (PSCs), CD200-/CD105- periosteal progenitor 1 (PP1) cells, and CD105+/CD200^variable^ periosteal progenitor 2 (PP2) cells. They claimed PSCs are broadly different from both their PP1 and PP2 derivatives, and PSCs displayed increased per-cell bone formation capacity compared to PP1/PP2 cells (Debnath et al., 2018). Our results showed that 89.1% cells were CD90+/CD105-, and within CD90+/Sca-1+ cells, 87.2% cells were CD45-/CD105-, which means the majority of our PDCs were CD90+/Sca-1+/CD105-/CD45-. Recent studies reported that DDR2- PDCs, Nestin+ PDCs and LepR+ PDCs possess multipotent and self-renewal abilities (Gao et al., 2019; Yang et al., 2020). Together, sorting a specific fraction of PDCs with highly pluripotency capability by using cell markers is promising and meaningful in bone tissue engineering and regeneration.

Osteoblast differentiation involves several stages including commitment of mesenchymal progenitors to the osteoblast lineage, osteoprogenitor proliferation, matrix maturation and mineralization. Wnt signaling was thought to stimulate early osteoblasts in their capacity to differentiate, whereas mature osteoblasts were strongly inhibited in their capacity to induce mineralization (Eijken et al., 2008). Some inhibitors of WNT signaling even have a positive role in osteoblast maturation, such as Dkk2 (Li et al., 2005). Previous study demonstrated that Wnt16 may act in a stage-dependent manner to regulate osteoblast differentiation. Wnt16 up-regulated Bmpr1b, Bmp7 and Enpp1, which mainly function in osteoblast differentiation stage, but rWnt16 down-regulated Alpl, Rspo2 and Ibsp, genes that are mainly involved in osteoblast maturation and mineralization stage (Sebastian et al., 2018). However, our result showed that Wnt16 accelerate both osteoblast differentiation and matrix mineralization of PDCs osteogenesis process. This controversy clearly needs further studies to settle down regarding to whether Wnt16 inhibit or promote mature osteoblast mineralization.

In the past years, researchers recognize that the effect of Wnt16-promoted osteogenesis in MSCs are coming from the activation of both canonical and non-canonical WNT signaling. Wnt16 activates Wnt/*β*-catenin, Wnt/Ca2+ and Wnt/PCP signaling via a specific G *α* subunits, which plays an orchestrating role in the downstream activity in osteoblasts (Gao et al., 2019; Hendrickx et al., 2020). Another paper suggested that non-canonical JNK pathway plays a key role in transcriptional activation of Wnt16 targets in osteoblasts (Sebastian et al., 2018). Our finding confirms that Wnt16 activates *β*-catenin signaling, yet suggests that Wnt16 may not trigger off Wnt/Ca2+ pathway but may indirectly inhibit Wnt/Ca2+ pathway by antagonizing Wnt5a. Reversely, Wnt5a is able to antagonize Wnt3a-induced *β*-catenin/TCF activity, reduce the stemness of hepatic progenitor cells, and promote hepatic differentiation of liver progenitors (Fan et al., 2017). Probably, Wnt16/*β*-catenin signaling and Wnt5a/Ca2+ pathway may exist a regulatory feedback loop. Obviously, additional studies are needed to demonstrate the intricate regulatory network among Wnt16, Wnt5a and their intracellular downstream pathways.

Our study has one limitation. The regenerated de novo bone formation in the present study is still unsatisfied. One reason may be the consequence of mixed cell population. Since our cells were not purified by cell markers such as CD90, CD105 and CD200, the un-purified cell population might contain fibroblasts, differentiated pre-osteoblasts/osteoblasts and were lack of progenitor cells, so that the transplant was not fully filled with new bone (Debnath et al., 2018; Sakai et al., 2011). On the other hand, albeit effective gene expression is desirable, yet to date the exogenous gene is often driven by only one constitutive promoter, whose effect is restricted to only one facet, such as osteogenesis, osteoclastogenesis, or angiogenesis. In contrast to the conventional gene therapy approach, the Bac-CRISPRa system, which is programmable and capable of activating multiple target genes, may be a promising technology in bone regeneration (Larouche & Aguilar, 2019). A recent study used CRISPRa-engineered BMSCs to activate Wnt10b and Foxc2, resulting in a remarkably improved calvarial bone healing (Hsu et al., 2020). Therefore, using cell markers to purified the primary PDCs and CRISPRs system therapy may further optimize bone healing.

## Conclusions

In summary, our data uncover Wnt16 act positively in PDCs osteogenesis. In vivo study proves overexpression of Wnt16 in PDCs could promote orthotopic de novo bone-like tissue formation in bone defect.

##  Supplemental Information

10.7717/peerj.10374/supp-1 Figure S1 Cell growth after knock down Wnt16 in PDCs Cell proliferation rate was determined by CCK-8 assay, no difference was found between control and Wnt16 knock down (kd) group.

10.7717/peerj.10374/supp-2 Figure S2 Wnt16 overexpressed in PDCs tagged with RFP (A–B) immunofluorescence images showed that the transfected PDCs were RFP positive in Wnt16 OE and control group. (C–D) FACS images showed 4.06% cells were RFP postive in blank group, while 26.5% and 22.1% cells were RFP positive in control and Wnt16 OE group.

10.7717/peerj.10374/supp-3 Supplemental Information 3 Figure 1 raw data

10.7717/peerj.10374/supp-4 Supplemental Information 4 Figure 2 raw data

10.7717/peerj.10374/supp-5 Supplemental Information 5 Figure 3 raw data

10.7717/peerj.10374/supp-6 Supplemental Information 6 Figure 4 raw data

10.7717/peerj.10374/supp-7 Supplemental Information 7 Raw data exported from the PCR ViiA 7 Real-Time PCR software applied for data analysis and preparation for the detailed investigation shown in Fig. 1B

10.7717/peerj.10374/supp-8 Supplemental Information 8 Raw data exported from the PCR ViiA 7 Real-Time PCR software applied for data analysis and preparation for the detailed investigation shown in Figs. 1F and 1H

10.7717/peerj.10374/supp-9 Supplemental Information 9 Raw data exported from the PCR ViiA 7 Real-Time PCR software applied for data analysis and preparation for the detailed investigation shown in Fig. 1C

10.7717/peerj.10374/supp-10 Supplemental Information 10 Raw data exported from the PCR ViiA 7 Real-Time PCR software applied for data analysis and preparation for the detailed investigation shown in Figs. 1F and 1H

10.7717/peerj.10374/supp-11 Supplemental Information 11 Raw data exported from the PCR ViiA 7 Real-Time PCR software applied for data analysis and preparation for the detailed investigation shown in Fig. 1C

10.7717/peerj.10374/supp-12 Supplemental Information 12 Raw data exported from the PCR ViiA 7 Real-Time PCR software applied for data analysis and preparation for the detailed investigation shown in Figs. 1F and 1H

## Additional Information and Declarations

### DNA Deposition

The following information was supplied regarding the deposition of DNA sequences: The mouse Wnt16 sequences are available at GenBank: NC_000072.6.

### Data Availability

The following information was supplied regarding data availability: Uncropped gels/blots are available at Figshare: Jin, Ying (2020): western blot. figshare. Figure. https://doi.org/10.6084/m9.figshare.12423740.v1. Raw data are available as Supplemental Files.
