# Supplementary figures and images for: Wnt16 signaling promotes osteoblast differentiation of periosteal derived cells in vitro and in vivo
Source: PeerJ. 2020 Nov 24;8:e10374. doi: 10.7717/peerj.10374 (PMC7694570; doi:10.7717/peerj.10374)

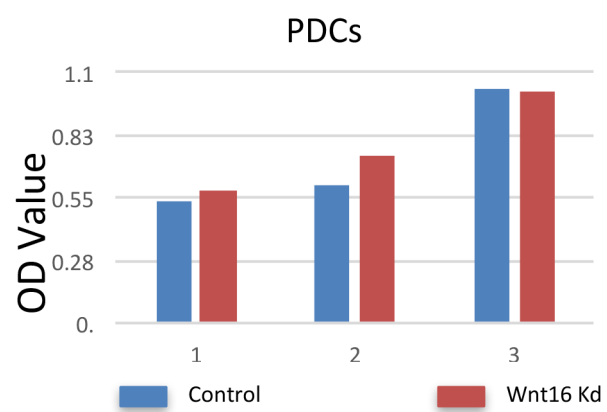

Supplement: Figure S1 — Cell proliferation rate was determined by CCK-8 assay, no difference was found between control and Wnt16 knock down (kd) group. [file peerj-08-10374-s001.pdf]

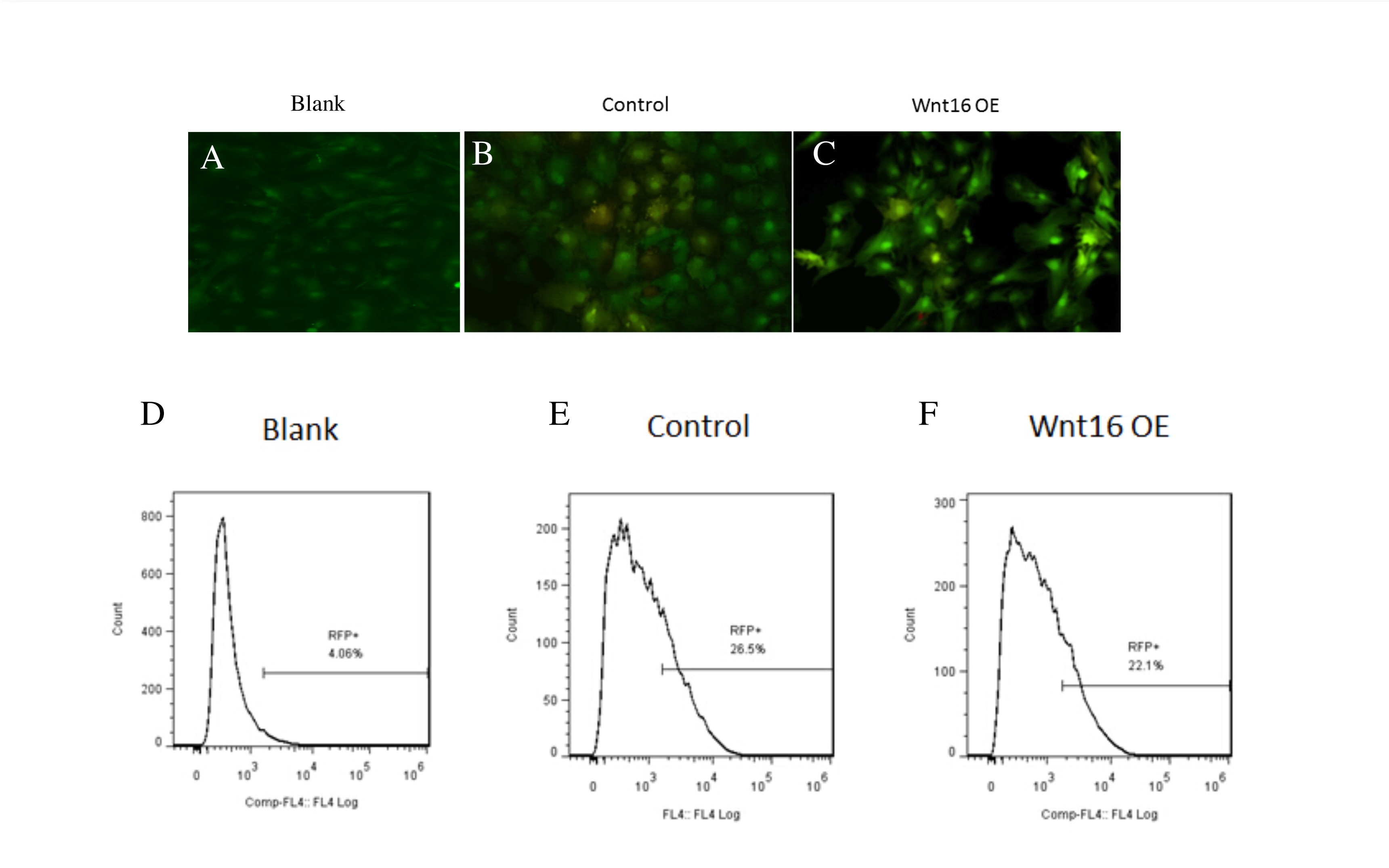

Supplement: Figure S2 — (A–B) immunofluorescence images showed that the transfected PDCs were RFP positive in Wnt16 OE and control group. (C–D) FACS images showed 4.06% cells were RFP postive in blank group, while 26.5% and 22.1% cells were RFP positive in control and Wnt16 OE group. [file peerj-08-10374-s002.jpg]
